# Linear dose-response relationship between vancomycin trough concentration and acute kidney injury in sepsis: effect modification by hyperlactatemia

**DOI:** 10.3389/fphar.2026.1817481

**Published:** 2026-07-29

**Authors:** Jing Tang, Daiyu Shen, Xun Zhou, Ling Xiao, Xinzhu Yuan, Bo Hu

**Affiliations:** 1 Department of Nephrology, Pangang Group General Hospital, Panzhihua, Sichuan, China; 2 Department of Laboratory Medicine, Pangang Group General Hospital, Panzhihua, Sichuan, China; 3 Department of Nephrology, Nanchong Central Hospital, Nanchong, China

**Keywords:** acute kidney injury, hyperlactatemia, sepsis, therapeutic drug monitoring, vancomycin

## Abstract

**Background:**

Vancomycin trough concentration (VTC) is commonly monitored to guide therapy in severe infections, yet its association with acute kidney injury (AKI) in septic patients remains inadequately characterized, particularly regarding the shape of the dose-response relationship and potential effect modifiers. This study aimed to investigate the relationship between initial VTC and AKI risk in sepsis, and to identify whether this association is modified by key clinical subgroups.

**Methods:**

We conducted a multicenter retrospective cohort study using the multicenter eICU Collaborative Research Database. Adults with sepsis who received intravenous vancomycin and had an initial trough concentration (VTC) measured were included; patients with acute kidney injury (AKI) diagnosed before the initial VTC measurement were excluded. The primary outcome was AKI during ICU stay, defined by KDIGO creatinine criteria. Multivariable logistic regression, generalized additive models, and subgroup analyses were used to evaluate the association between initial VTC and AKI, testing for interactions with prespecified covariates. Aminoglycoside use was rare (n = 3, 0.3%) and was therefore not included in the analysis.

**Results:**

Among 946 eligible patients, the overall AKI incidence was 9.7%. After multivariable adjustment, each 1 mg/L increase in initial VTC was associated with 6% higher odds of AKI (adjusted OR 1.06, 95% CI 1.02–1.11, *P* = 0.002). Patients with VTC >20 mg/L had a 3.5-fold higher risk compared with those with VTC ≤10 mg/L. The dose-response relationship was approximately linear (edf = 1.00, *P* = 0.002), with limited evidence for a distinct threshold. A significant interaction was observed for lactate level (*P* for interaction = 0.025): in patients with lactate >2.0 mmol/L, each 1 mg/L increase in VTC was associated with a 13% higher odds of AKI (aOR 1.13, 95% CI 1.05–1.21, *P* = 0.001), whereas no significant association was found in those with lactate ≤2.0 mmol/L (aOR 1.03, 95% CI 0.98–1.08, *P* = 0.258).

**Conclusion:**

In septic patients, higher initial VTC is independently and linearly associated with increased AKI risk without a discrete safety threshold. This association is significantly stronger in patients with hyperlactatemia, generating the hypothesis that lactate level identifies a subgroup with heightened susceptibility to vancomycin-associated nephrotoxicity, which requires prospective validation.

## Introduction

Vancomycin, a glycopeptide antimicrobial agent, is a first-line treatment for serious Gram-positive infections such as methicillin-resistant *Staphylococcus aureus* (MRSA) in the intensive care setting (Moellering, 2006). While the area under the concentration-time curve over 24 h (AUC_0_-_24_/MIC) ratio is the pharmacokinetic/pharmacodynamic (PK/PD) index best correlated with efficacy (Rybak et al., 2009; Álvarez et al., 2016), the therapeutic drug monitoring (TDM) of vancomycin in clinical practice has been predominantly guided by the pre-dose trough concentration (VTC) for decades, based on historical pragmatism (van Hal et al., 2013; Rybak, 2006). This entrenched reliance on a pharmacokinetically limited surrogate persists despite growing recognition of its pharmacokinetic limitations and the contemporary availability of Bayesian tools that facilitate AUC-guided dosing with minimal blood sampling (Rybak et al., 2020; Alnezary et al., 2023).

Critically, this paradigm creates a direct clinical dilemma: clinicians are making dose adjustments based on a metric (VTC) whose relationship with the desired outcome (efficacy) is weak and inconsistent (Hou et al., 2021), yet whose association with a major harm (nephrotoxicity) appears strong and dose-dependent (Kullar et al., 2011). Therefore, precisely because trough-based monitoring remains a widespread clinical reality, quantifying the real-world renal risk associated with specific trough levels—especially the initial VTC that sets the early therapeutic course—is of immediate practical importance for patient safety.

This risk quantification is most urgent in patients with sepsis, a population at high baseline risk for acute kidney injury (AKI) due to multifactorial insults (Sun et al., 2023; Wang et al., 2022). Furthermore, the severe pathophysiology of sepsis may alter vancomycin pharmacokinetics and renal susceptibility (Medellín-Garibay et al., 2017; Heffernan et al., 2019), potentially modifying the VTC-AKI relationship in ways that are currently unknown. Whether the renal risk signaled by the initial VTC is uniform or varies across key clinical subgroups remains unexplored.

It remains unclear whether the initial VTC, a key metric guiding early dosing, carries a differential risk for acute kidney injury across clinically distinct subgroups of septic patients. This study aimed to examine the association between initial VTC and AKI in sepsis, to characterize the shape of the dose-response relationship, and to identify potential effect modifiers of this association. Furthermore, whether the VTC-AKI association is modified by clinical biomarkers such as lactate remains unknown.

## Materials and Methods

### Data source and study population

This retrospective cohort study utilized data from the eICU Collaborative Research Database (eICU-CRD, version 2.0), a multi-center critical care database containing de-identified clinical records of over 200,000 ICU admissions across the United States between 2014 and 2015 (Pollard et al., 2018). The database is publicly available, and its use for this analysis was approved by the institutional review board of the Massachusetts Institute of Technology (Cambridge, MA, United States of America). Informed consent was waived due to the de-identified nature of the data. One author, who completed the mandated eICU-CRD training program (Certification Number: 63668742), performed the data extraction. The conduct of this study adhered to the ethical principles of the Declaration of Helsinki, and its reporting follows the Strengthening the Reporting of Observational Studies in Epidemiology (STROBE) guidelines. Access to the eICU-CRD is available to qualified researchers through an official application process via the project website (https://eicu-crd.mit.edu/about/eicu/).

### Patient selection

**FIGURE 1:**
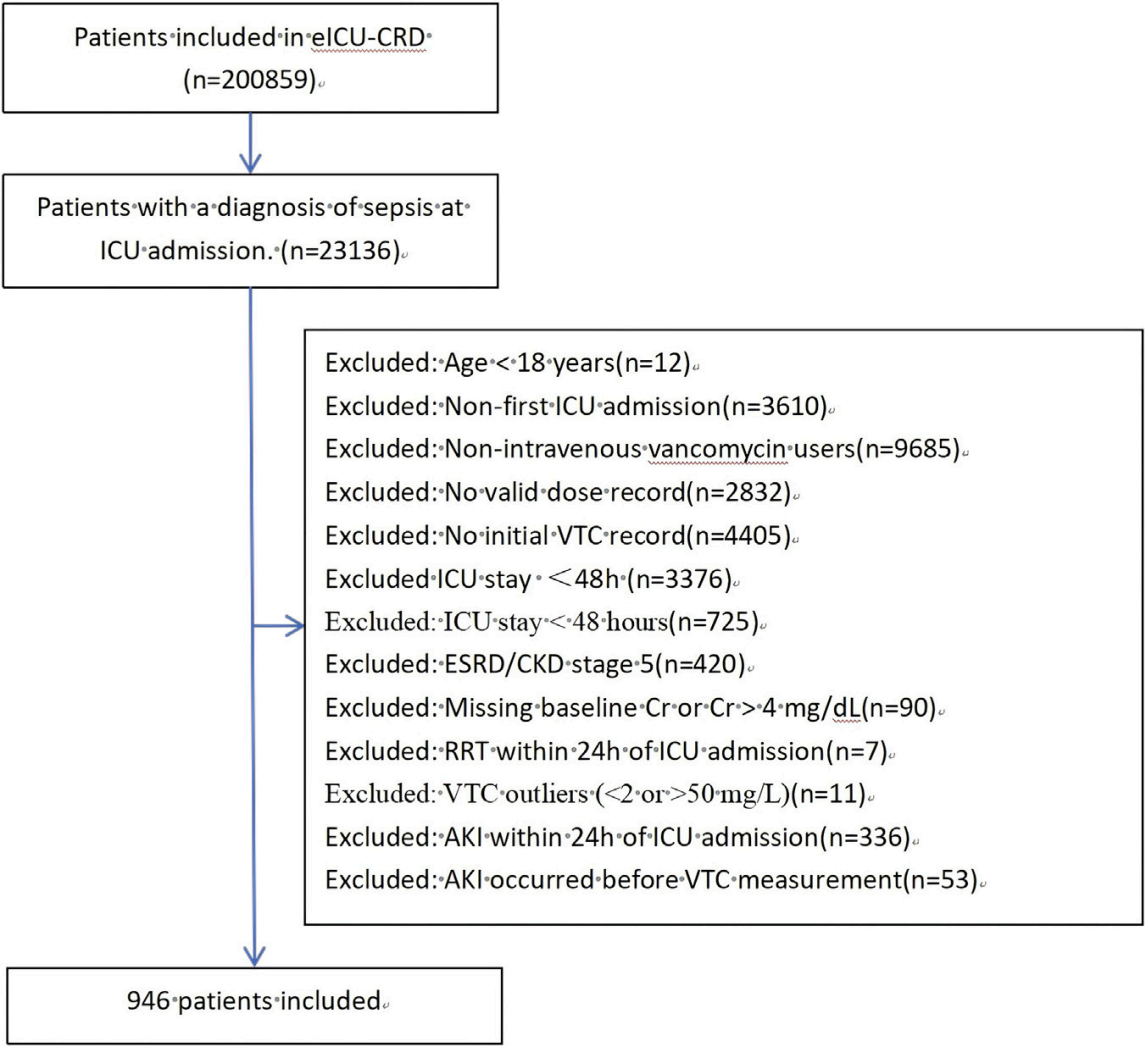
Flow diagram of patient selection. Final cohort: 946 sepsis patients from 200,859 eICU patients. See Methods for exclusion criteria. Abbreviations: eICU-CRD, eICU Collaborative Research Database; VTC, vancomycin trough concentration; AKI, acute kidney injury; Cr, creatinine; ESRD, end-stage renal disease; CKD, chronic kidney disease; RRT, renal replacement therapy.

Adult patients (aged ≥18 years) with sepsis (defined according to Sepsis-3 criteria) who received intravenous vancomycin during their first ICU admission were identified from the eICU-CRD. Eligible patients were required to have at least one documented vancomycin trough concentration. Patients were excluded if they met any of the following criteria:no valid vancomycin dose record, no initial trough concentration, ICU stay <48 h, end-stage renal disease or CKD stage 5, baseline serum creatinine >4 mg/dL or missing, renal replacement therapy within 24 h of ICU admission, trough concentration <2 or >50 mg/L, acute kidney injury (AKI) diagnosed within 24 h of ICU admission, or AKI occurring before the initial trough measurement. After sequential application of these criteria, a final cohort of 946 patients was included. A detailed patient selection process is illustrated in Figure 1.

## Exposure, outcomes, and covariates

### Exposure

The primary exposure was the initial vancomycin trough concentration (VTC, mg/L), defined as the first documented trough level after ICU admission. In the eICU database, trough values were defined as those drawn within 30 min before the next scheduled vancomycin dose, consistent with the institutional standard. While trough concentrations were extracted from clinical documentation and laboratory timing, the exact sampling time relative to the last dose could not be verified in the eICU-CRD. Based on established clinical thresholds, VTC was categorized into three groups: low (≤10 mg/L), intermediate (>10 to ≤20 mg/L), and high (>20 mg/L).

### Outcomes

The primary outcome was acute kidney injury (AKI) during the ICU stay, defined according to the serum creatinine-based criteria of the Kidney Disease: Improving Global Outcomes (KDIGO) guidelines. Urine output criteria were not applied due to unreliable data availability. Baseline serum creatinine was defined as the first value measured within 7 days prior to ICU admission; if unavailable, the first value within 24 h after admission was used instead. Patients without any creatinine measurement in either window were excluded. AKI was defined as an increase in serum creatinine of ≥0.3 mg/dL within 48 h or an increase to ≥1.5 times baseline within 7 days. To ensure temporal precedence, only AKI events occurring after the initial VTC measurement were counted.

### Covariates

Covariates were selected based on clinical relevance and prior literature, and were assessed at or within the first 24 h of ICU admission. Demographic variables included age, sex, ethnicity, and body mass index (BMI). Baseline severity was assessed using the Sequential Organ Failure Assessment (SOFA) score; the Glasgow Coma Scale (GCS) score and Acute Physiology and Chronic Health Evaluation (APACHE) IV score were also collected. Laboratory variables included albumin, lactate, and platelet count. Comorbidities included chronic kidney disease (CKD). The primary source of infection was categorized as pulmonary, renal/urinary tract, gastrointestinal, cutaneous/soft tissue, or other. Nephrotoxic medication exposure (vasopressor use and diuretic use) was restricted to the 7 days prior to or during vancomycin therapy to avoid misclassification of home medications. Aminoglycoside use was rare (n = 3, 0.3%) and was therefore not included in the analysis.

Due to the limited number of AKI events (n = 92), the primary multivariable model adjusted for a core set of covariates: age, gender, BMI, SOFA score, vasopressor use, CKD, albumin, lactate, platelet count, and diuretic use. This corresponds to an events-per-variable (EPV) ratio of approximately 9.2, slightly below the conventional threshold of 10. However, the absence of significant multicollinearity (all VIF <5; Supplementary Table S7) and the consistency of effect estimates across sensitivity analyses support the stability of our regression model.

Given the limited number of events, GCS and APACHE IV scores were not included in the primary model to avoid overfitting; they were instead reserved for sensitivity analyses (Supplementary Tables S5, S6).

### Statistical analysis

Continuous variables are presented as median (interquartile range, IQR) based on their non-normal distribution, as confirmed by the Shapiro-Wilk test. Categorical variables are expressed as numbers (percentages). Differences among the three VTC groups (Low, Intermediate, High) were assessed using the Kruskal–Wallis test for continuous variables and the Chi-square or Fisher’s exact test for categorical variables, as appropriate.

The association between initial VTC and AKI was evaluated using univariable and multivariable logistic regression. Covariates were selected *a priori* based on clinical knowledge. Three models were constructed: a crude model (unadjusted); Model 1 (adjusted for age, gender, and BMI); and Model 2, the primary model (adjusted for age, gender, BMI, SOFA score, vasopressor use, CKD, albumin, lactate, platelet count, and diuretic use). Collinearity diagnostics showed no significant multicollinearity among the covariates (all VIF <5; Supplementary Tables S7). GCS and APACHE IV scores were not included in the primary model due to the limited number of AKI events (n = 92) and were reserved for sensitivity analyses (Supplementary Tables S5, S6).

To examine the dose-response relationship, a generalized additive model (GAM) with a cubic regression spline was fitted, adjusting for all covariates in Model 2. A two-piecewise regression model was used to identify potential threshold effects, with the optimal knot selected by minimizing the Akaike information criterion; a likelihood ratio test compared the two-piecewise model with the linear model.

Subgroup analyses were performed to evaluate effect modification by clinically relevant variables (age, gender, BMI, SOFA score, lactate, platelet count, vasopressor use, diuretic use, and albumin). Interaction terms between each subgroup variable and initial VTC were introduced into the fully adjusted model, with *P*-values obtained via likelihood ratio tests.

Sensitivity analyses, including multiple imputation (Supplementary Tables S2, S3), hospital clustering (Supplementary Tables S4), and additional adjustment for GCS and APACHE IV scores (Supplementary Tables S5, S6), confirmed the robustness of the findings. All tests were two-sided, with *P* < 0.05 considered significant. Analyses were performed using R version 4.2.0.

## Results

### Baseline characteristics

Of the 946 sepsis patients included, 92 (9.7%) developed AKI and 74 (7.8%) died within 28 days. Compared with the low (≤10 mg/L, n = 337) and intermediate (10–20 mg/L, n = 424) VTC groups, patients in the high VTC group (>20 mg/L, n = 185) had higher BMI and SOFA scores, more frequent vasopressor use, and lower albumin levels (all *P* < 0.05). AKI incidence increased stepwise across VTC groups (6.5%, 9.9%, and 15.1%, *P* = 0.006), as did 28-day mortality (5.3%, 6.8%, and 14.6%, *P* < 0.001). Other baseline characteristics, including age, gender, ethnicity, comorbidities, and infection source, were comparable across groups (Table 1). Baseline comparisons of GCS and APACHE IV scores across VTC groups are provided in Supplementary Tables S8.

**TABLE 1:** Baseline characteristics and clinical outcomes of patients with sepsis stratified by initial vancomycin trough concentration.

Characteristic	​	Initial VTC			*P* Value
	Entire population	Low trough (≤10 mg/L)	Intermediate trough (10–20 mg/L)	High trough (>20 mg/L)	
	(N = 946)	(N = 337)	(N = 424)	(N = 185)	
Exposure					
Initial VTC, mg/L	12.85 (8.62–18.40)	7.50 (6.00–8.90)	14.20 (12.20–16.80)	24.10 (21.90–28.40)	<0.001
Demographic data					
Age, years	64.00 (53.00–74.00)	63.00 (52.00–74.00)	65.00 (54.00–75.00)	62.00 (52.00–73.00)	0.133
Gender, n (%)	​	​	​	​	0.590
Female	421 (44.5%)	154 (45.7%)	181 (42.7%)	86 (46.5%)	​
Male	525 (55.5%)	183 (54.3%)	243 (57.3%)	99 (53.5%)	​
Ethnicity, n (%)	​	​	​	​	0.736
Caucasian	716 (75.7%)	244 (72.4%)	329 (77.6%)	143 (77.3%)	​
African American	109 (11.5%)	44 (13.1%)	43 (10.1%)	22 (11.9%)	​
Hispanic	67 (7.1%)	31 (9.2%)	26 (6.1%)	10 (5.4%)	​
Asian	18 (1.9%)	6 (1.8%)	9 (2.1%)	3 (1.6%)	​
Native American	15 (1.6%)	4 (1.2%)	7 (1.7%)	4 (2.2%)	​
Other/Unknown	21 (2.2%)	8 (2.4%)	10 (2.4%)	3 (1.6%)	​
BMI, kg/m^2^	26.58 (22.38–31.87)	24.51 (21.22–29.82)	26.62 (22.77–31.48)	29.24 (24.62–36.53)	<0.001
Severity score					
SOFA score	4.00 (2.00–6.00)	4.00 (2.00–6.00)	4.00 (2.00–6.00)	5.00 (3.00–7.00)	0.004
Comorbidities					
Chronic kidney disease, n (%)	164 (17.3%)	63 (18.7%)	69 (16.3%)	32 (17.3%)	0.681
Sepsis source of infection	​	​	​	​	0.210
*Pulmonary*	466 (49.3%)	180 (53.4%)	206 (48.6%)	80 (43.2%)	​
*Renal/UTI*	156 (16.5%)	52 (15.4%)	67 (15.8%)	37 (20.0%)	​
*GI*	89 (9.4%)	28 (8.3%)	40 (9.4%)	21 (11.4%)	​
*Cutaneous/soft tissue*	104 (11.0%)	35 (10.4%)	47 (11.1%)	22 (11.9%)	​
*Gynecologic*	1 (0.1%)	0 (0.0%)	0 (0.0%)	1 (0.5%)	​
Laboratory values					
Albumin, g/dL	2.40 (2.00–2.80)	2.50 (2.10–2.90)	2.40 (2.00–2.70)	2.30 (1.80–2.70)	<0.001
Lactate, mmol/L	1.60 (1.10–2.60)	1.65 (1.08–2.60)	1.60 (1.10–2.50)	1.70 (1.20–2.80)	0.578
Platelets, ×10^9^/L	188.00 (121.00–269.25)	171.00 (119.00–264.00)	196.00 (125.00–280.00)	188.00 (116.25–259.75)	0.116
Nephrotoxic medications					
Vasopressor use, n (%)	343 (36.3%)	120 (35.6%)	140 (33.0%)	83 (44.9%)	0.019
Diuretic use, n (%)	440 (46.5%)	145 (43.0%)	202 (47.6%)	93 (50.3%)	0.233
Aminoglycoside use, n (%)	3 (0.3%)	2 (0.6%)	1 (0.2%)	0 (0.0%)	0.604
ACEI/ARB use, n (%)	77 (8.1%)	36 (10.7%)	35 (8.3%)	6 (3.2%)	0.012
NSAID use, n (%)	49 (5.2%)	24 (7.1%)	16 (3.8%)	9 (4.9%)	0.115
Outcomes					
AKI occurrence, n (%)	92 (9.7%)	22 (6.5%)	42 (9.9%)	28 (15.1%)	0.006
28-day mortality, n (%)	74 (7.8%)	18 (5.3%)	29 (6.8%)	27 (14.6%)	<0.001

Data are presented as mean ± SD, or median (IQR) based on normality test. Group comparisons: Kruskal–Wallis test for continuous variables, Chi-square/Fisher’s exact test for categorical variables. Nephrotoxic medication exposure, including vasopressor use, diuretic use, ACEI/ARB, and NSAID, was assessed during the 7 days prior to or during vancomycin therapy.

Abbreviations: AKI, acute kidney injury; BMI, body mass index; CKD, chronic kidney disease; IQR, interquartile range; SOFA, Sequential Organ Failure Assessment; VTC, vancomycin trough concentration; ACEI, angiotensin-converting enzyme inhibitor; ARB, angiotensin receptor blocker; NSAID, non-steroidal anti-inflammatory drug.

Missing data: BMI (1.5%), albumin (27.6%), lactate (36.0%), platelets (2.7%).

### Association of initial vancomycin trough concentration with AKI

Univariable analysis showed that higher initial VTC, lower albumin, higher lactate, higher platelet count, and vasopressor and diuretic use were significantly associated with AKI (all P < 0.05, Supplementary Tables S1). Age was inversely associated with AKI (*P* < 0.001).

Multivariable logistic regression confirmed an independent positive association (Table 2). In the fully adjusted model, each 1 mg/L increase in initial VTC was associated with a 6% higher odds of AKI (aOR 1.06, 95% CI 1.02–1.11, *P* = 0.002). Compared with the low VTC group (≤10 mg/L), the high VTC group (>20 mg/L) had a 3.5-fold increased risk of AKI (aOR 3.52, 95% CI 1.46–8.45, *P* = 0.005). The intermediate group (10–20 mg/L) showed a trend toward increased risk that did not reach statistical significance (aOR 2.27, 95% CI 0.90–5.74, *P* = 0.083). Nevertheless, the overall pattern across VTC groups—with AKI incidence increasing stepwise from 6.5% to 9.9% to 15.1%—is consistent with a dose-response relationship.

**TABLE 2:** Multivariable logistic regression analysis of the association between initial VTC and AKI.

Exposure	Crude model OR (95% CI)	*P* Value	Model 1 OR (95% CI)	*P* Value	Model 2 OR (95% CI)	*P* Value
Initial VTC (per 1 mg/L)	1.07 (1.03–1.10)	<0.001	1.04 (1.02–1.07)	0.002	1.06 (1.02–1.11)	0.002
VTC tertiles						
Low (≤10 mg/L)	References	​	References	​	References	​
Middle (>10 to ≤20 mg/L)	2.54 (1.05–6.13)	0.038	1.68 (0.91–3.12)	0.099	2.27 (0.90–5.74)	0.083
High (>20 mg/L)	4.41 (1.94–10.02)	<0.001	2.66 (1.49–4.74)	<0.001	3.52 (1.46–8.45)	0.005

Data are presented as OR (95% CI). Crude model: unadjusted. Model 1: adjusted for age, gender, and BMI., Model 2: adjusted for age, gender, BMI, SOFA, score, vasopressor use; CKD, albumin, lactate, platelet count, and diuretic use. Abbreviations: VTC, vancomycin trough concentration; AKI, acute kidney injury; BMI, body mass index; SOFA, sequential organ failure assessment; CKD, chronic kidney disease.

### Dose-response relationship between initial VTC and AKI

**FIGURE 2:**
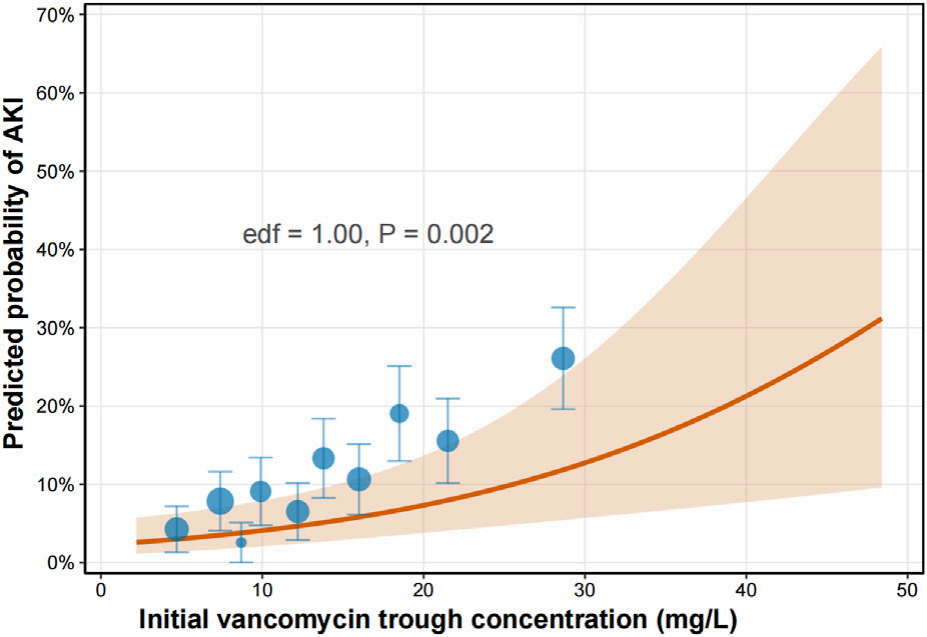
Dose-response relationship between initial VTC and AKI. Predicted probability from a GAM adjusted for age, gender, BMI, SOFA, vasopressor use, CKD, albumin, lactate, platelet count, and diuretic use. Solid line: predicted probability; shaded area: 95% CI; dots: observed AKI rates by VTC deciles (point size proportional to sample size). Linear association: edf = 1.00, *P* = 0.002. Abbreviations: VTC, vancomycin trough concentration; AKI, acute kidney injury; GAM, generalized additive model; edf, effective degrees of freedom. The shaded area represents 95% confidence intervals, which widen at higher VTC levels (>30 mg/L) due to sparse data.

A generalized additive model revealed an approximately linear relationship between initial VTC and AKI (effective degrees of freedom (edf) = 1.00, *P* = 0.002; Figure 2). It should be noted that the confidence intervals widened considerably at VTC levels >30 mg/L due to sparse observations in this range, indicating greater uncertainty in the estimated risk at very high concentrations. A two-piecewise regression model identified a potential threshold at 24 mg/L (Table 3). Below this threshold, each 1 mg/L increase in initial VTC was associated with a 9% higher odds of AKI (aOR 1.09, 95% CI 1.02–1.15, *P* = 0.007); above 24 mg/L, the association was attenuated and no longer significant (aOR 1.02, 95% CI 0.93–1.12, *P* = 0.623). However, the likelihood ratio test did not favor the two-piecewise model over the linear model (*P* = 0.371), indicating that a distinct threshold was not robustly supported.

**TABLE 3:** Threshold effect analysis of the relationship between the initial VTC and AKI using a two-piecewise regression model.

Models	AKI or (95%CI)	*P*-value
Model I		
Linear effect	1.06 (1.02–1.11)	0.002
Model II		
Knot (K)	24	​
Effect 1 (<K)	1.09 (1.02–1.15)	0.007
Effect 2 (>K)	1.02 (0.93–1.12)	0.623
Likelihood-ratio test	​	0.371

Data are presented as OR (95% CI). Model I: linear effect model. Model II: two-piecewise regression model with optimal knot (K = 24 mg/L) determined by minimizing AIC., Effect 1: slope for VTC < K. Effect 2: slope for VTC > K. Likelihood ratio test compares two-piecewise model vs. linear model. All models were adjusted for the 10 covariates (age, gender, BMI, SOFA, vasopressor use; CKD, albumin, lactate, platelet count, and diuretic use). Abbreviations: VTC, vancomycin trough concentration; AKI, acute kidney injury; AIC, akaike information criterion.

### Subgroup and interaction analyses

The positive association between initial VTC and AKI was generally consistent across most prespecified strata (Table 4). A significant interaction was observed for lactate level (*P* for interaction = 0.025). In patients with lactate >2.0 mmol/L, each 1 mg/L increase in initial VTC was associated with a 13% higher odds of AKI (aOR 1.13, 95% CI 1.05–1.22, *P* < 0.001), whereas no significant association was found in those with lactate ≤2.0 mmol/L (aOR 1.03, 95% CI 0.98–1.08, *P* = 0.259). No significant interactions were observed for age, gender, BMI, SOFA score, platelet count, vasopressor use, diuretic use, or albumin (all *P* for interaction >0.05).

**TABLE 4:** Subgroup analysis of the association between initial VTC and AKI.

Subgroup	Level	N	AKI	Or (95% CI)	*P* Value	*P* For interaction
Overall	​	452	52	1.06 (1.02–1.11)	0.002	​
Age (years)	​	​	​	​	​	0.537
	<65	249	39	1.08 (1.03–1.13)	0.002	​
	≥65	203	13	1.06 (0.98–1.14)	0.130	​
Gender	​	​	​	​	​	0.861
	Female	187	22	1.07 (1.01–1.13)	0.021	​
	Male	265	30	1.06 (1.00–1.12)	0.056	​
BMI (kg/m^2^)	​	​	​	​	​	0.967
	<30	304	28	1.06 (1.01–1.12)	0.016	​
	≥30	148	24	1.08 (1.01–1.15)	0.034	​
SOFA score	​	​	​	​	​	0.215
	<6	266	29	1.09 (1.03–1.15)	0.004	​
	≥6	186	23	1.05 (0.99–1.11)	0.094	​
Lactate (mmol/L)	​	​	​	​	​	0.025
	≤2.0	279	27	1.03 (0.98–1.08)	0.258	​
	>2.0	173	25	1.13 (1.05–1.21)	0.001	​
Platelets (×10^9^/L)	​	​	​	​	​	0.154
	≥150	272	23	1.04 (0.99–1.09)	0.123	​
	<150	180	29	1.10 (1.03–1.18)	0.007	​
Vasopressor use	​	​	​	​	​	0.238
	No	261	23	1.10 (1.02–1.17)	0.009	​
	Yes	191	29	1.05 (1.00–1.10)	0.060	​
Diuretic use	​	​	​	​	​	0.877
	No	237	20	1.07 (1.00–1.13)	0.039	​
	Yes	215	32	1.06 (1.01–1.12)	0.030	​
Albumin (g/dL)	​	​	​	​	​	0.774
	≥3.0	74	7	0.99 (0.87–1.13)	0.887	​
	<3.0	378	45	1.08 (1.04–1.12)	<0.001	​

Abbreviations: VTC, vancomycin trough concentration; AKI, acute kidney injury; OR, odds ratio; CI, confidence interval; BMI, body mass index; SOFA, Sequential Organ Failure Assessment. All models were adjusted for 10 covariates (age, gender, BMI, SOFA, vasopressor use; CKD, albumin, lactate, platelet count, diuretic use), except the stratification variable. *P* for interaction was calculated using the likelihood ratio test comparing models with and without the interaction term. OR, represents the change in AKI, odds per 1 mg/L increase in initial VTC., Results are based on complete-case analysis (N = 452, AKI, events = 52). Missing data: lactate 36.0%, albumin 27.6%, BMI, 1.5%, platelets 2.7%. Albumin cutoff: 3.0 g/dL. SOFA, cutoff: 6. A significant interaction was observed for lactate (*P* = 0.025).

### Sensitivity analyses

The positive association between initial VTC and AKI was robust across sensitivity analyses, including multiple imputation (Supplementary Tables S2, S3), hospital clustering (Supplementary Tables S4), and severe AKI analysis (Supplementary Tables S9, S10). Multiple imputation yielded a consistent estimate (aOR 1.03, 95% CI 1.00–1.06, *P* = 0.030), hospital clustering did not materially change the results (aOR 1.06, 95% CI 1.02–1.11, *P* = 0.002), and the severe AKI analysis remained significant (aOR 1.06, 95% CI 1.00–1.12, *P* = 0.036). The threshold effect was attenuated in all sensitivity analyses (all likelihood ratio test *P* > 0.15). Collectively, these analyses support the robustness of a positive, approximately linear dose-response relationship.

## Discussion

In this multicenter retrospective study of 946 patients with sepsis, we identified a significant and independent association between initial vancomycin trough concentration (VTC) and acute kidney injury (AKI). After adjusting for 10 prespecified covariates, each 1 mg/L increase in initial VTC was associated with a 6% higher odds of AKI. Patients in the high VTC group (>20 mg/L) had a more than threefold increased risk compared with the low VTC group (≤10 mg/L), demonstrating a concentration-dependent relationship. The dose-response relationship was linear, with limited evidence for a distinct threshold. Notably, the association was significantly stronger in patients with elevated lactate levels. Sensitivity analyses confirmed the robustness of these findings. Of note, current consensus guidelines recommend AUC-guided vancomycin monitoring over trough-based monitoring. Our findings are most relevant to clinical settings where trough-based monitoring remains common. Where AUC monitoring is available, it should be preferred for optimizing both efficacy and safety.

Our findings corroborate and enhance the existing body of evidence regarding vancomycin-associated nephrotoxicity. The landmark meta-analysis by van Hal et al. demonstrated that trough concentrations ≥15 mg/L were associated with a 2.67-fold higher odds of nephrotoxicity compared with lower troughs (van Hal et al., 2013). Our results are consistent with this magnitude of effect: in the fully adjusted model, patients with initial VTC >20 mg/L had a 3.5-fold higher odds of AKI compared with those with VTC ≤10 mg/L (aOR 3.52, 95% CI 1.46–8.45, *P* = 0.005). Zamoner et al. prospectively studied 63 critically ill septic patients and found that vancomycin concentrations >17.53 mg/L between days 2–4 of therapy predicted AKI, preceding diagnosis by at least 48 h (Zamoner et al., 2022). Bao et al., in a large analysis of 1,959 ICU patients from the MIMIC-IV database, identified each 1 mg/L increase in vancomycin trough concentration as an independent predictor of AKI (OR 1.02, 95% CI 1.00–1.04) (Bao et al., 2024). Our study found a slightly larger effect size (aOR 1.06 per 1 mg/L, 95% CI 1.02–1.11, *P* = 0.002), which may reflect our exclusive focus on sepsis patients, who may be more susceptible to vancomycin-induced kidney injury due to the “second-hit” priming of the renal tubules.

Despite the consistent finding of concentration-dependent nephrotoxicity, the shape of the dose-response relationship differs between our study and many previous reports. Numerous studies have identified specific thresholds for vancomycin-associated AKI: Yang et al. reported a trough cut-off of 14.55 mg/L in a quantitative meta-analysis (Yang et al., 2024). Hong et al. identified vancomycin trough concentration >20.0 μg/mL as an independent risk factor for AKI in overweight patients (OR 6.251, 95% CI 2.275–17.180) (Hong et al., 2024) and Zamoner et al. reported a threshold of 17.53 mg/L in critically ill septic patients (Zamoner et al., 2022). In contrast, our analysis revealed an approximately linear relationship between initial VTC and AKI risk. The generalized additive model showed no significant deviation from linearity (Figure 2), and the likelihood ratio test did not support a non-linear model over a linear model (Table 3). This linear pattern indicates that in septic patients, AKI risk increases progressively with rising vancomycin exposure rather than emerging abruptly at a specific concentration. Accordingly, our data do not support a discrete safety threshold and suggest a continuous risk assessment approach in this population.

The modifying effect of lactate on the VTC-AKI association observed in our study (*P* for interaction = 0.025) is supported by existing literature. Lacave et al., in a cohort of 107 critically ill patients receiving continuous high-dose vancomycin, identified peak serum lactate as a significant predictor of AKI (*P* < 0.01) and found that the cumulative number of organ failures—including circulatory failure—was independently associated with AKI (OR 2.63, 95% CI 1.42–5.31, *P* < 0.01) (Lacave et al., 2017). These findings align with our observation that hyperlactatemia (>2.0 mmol/L) identifies patients in whom the VTC-AKI association is substantially stronger (aOR 1.13 per 1 mg/L, 95% CI 1.05–1.22) compared with those with lactate ≤2.0 mmol/L (aOR 1.03, 95% CI 0.98–1.08). Mechanistically, elevated lactate reflects microcirculatory dysfunction and tissue hypoperfusion (Suetrong and Walley, 2016; Schenz et al., 2021), which may reduce vancomycin clearance and increase tubular exposure (Betrie et al., 2023). Furthermore, a preclinical study demonstrated that clinically relevant vancomycin concentrations induce anabolic glucose reactions in renal epithelial cells, resulting in increased lactate production (Lagies et al., 2021). This suggests a potential feed-forward loop wherein vancomycin accumulation exacerbates local metabolic stress, amplifying tubular injury.

Vancomycin nephrotoxicity begins with its selective accumulation in proximal tubular epithelial cells. Megalin, a multiligand endocytic receptor on the apical membrane, mediates cellular uptake of vancomycin. Megalin blockade with cilastatin or genetic deletion protects against tubular injury, establishing this pathway as critical for vancomycin toxicity (Han et al., 2022). Once internalized, vancomycin induces oxidative stress in proximal tubular cells (Alosaimy et al., 2024). In septic patients, the tubular epithelium is already primed by microcirculatory dysfunction, inflammation, and mitochondrial stress (Iba et al., 2024; Ren et al., 2026). Vancomycin thus acts as a second hit on an already vulnerable substrate. This framework makes the lactate interaction observed in our study biologically coherent: elevated lactate marks microcirculatory compromise, and higher vancomycin exposure in these patients imposes additional oxidative burden more likely to cross the threshold into clinically detectable AKI (Li et al., 2025; Huang et al., 2026). Clinically, these findings suggest that hyperlactatemic septic patients warrant more vigilant vancomycin monitoring and dose adjustment, and support a continuous risk assessment approach over fixed concentration thresholds.

Several limitations should be acknowledged. First, the retrospective observational design precludes causal inference. Although we excluded patients with AKI diagnosed before the initial VTC measurement, we cannot rule out reverse causation—subclinical AKI or early renal function decline prior to trough measurement may have contributed to higher VTC values. Additionally, the exact timing of trough measurements relative to vancomycin dosing could not be independently verified, introducing non-differential misclassification that would bias the association toward the null. Second, selection bias is possible: requiring ICU stay ≥48 h excluded patients with very early death (18.2% mortality in excluded patients vs. 7.8% in the included cohort), limiting generalizability to those with rapid recovery or early mortality. Third, AKI was defined using creatinine criteria only, as urine output data were unreliable, potentially underdiagnosing milder AKI. Baseline creatinine was estimated from values within 7 days prior to ICU admission or within the first 24 h post-admission if unavailable. This may introduce bidirectional bias; early post-admission values can be diluted by fluid resuscitation, lowering baseline creatinine and inflating AKI incidence. However, such misclassification is likely non-differential with respect to vancomycin exposure. Fourth, residual confounding may persist due to unmeasured variables (e.g., vancomycin dosing intensity in mg/kg, duration of therapy, fluid balance, other nephrotoxins such as piperacillin-tazobactam) and the absence of microbiological outcomes to assess the efficacy side of the therapeutic balance. While we adjusted for the achieved trough concentration, we did not account for weight-based dosing or treatment duration, which may influence both vancomycin exposure and AKI risk. Fifth, our findings are derived exclusively from sepsis patients in the eICU-CRD; therefore, generalizability to non-sepsis populations or other healthcare settings requires further validation. Additionally, the events-per-variable ratio of approximately 9.2, while slightly below the conventional threshold of 10, may limit the number of covariates we could adjust for; however, collinearity diagnostics and sensitivity analyses support the robustness of our estimates.

## Conclusion

In summary, for sepsis patients receiving vancomycin, a higher initial trough concentration is independently and linearly associated with an increased risk of acute kidney injury, without a discrete safety threshold. These hypothesis-generating findings suggest heightened susceptibility in patients with hyperlactatemia (lactate >2.0 mmol/L), but prospective validation is required before any clinical recommendation can be made.

## Data Availability

Publicly available datasets were analyzed in this study. This data can be found here: The datasets presented in this study can be found in online repositories. The name of the repository and the link can be found below: eICU Collaborative Research Database (eICU-CRD), available at: https://eicu-crd.mit.edu/about/eicu/. Data access requires registration and training in accordance with the database’s data use agreement.
