# Supplementary material for: Linear dose-response relationship between vancomycin trough concentration and acute kidney injury in sepsis: effect modification by hyperlactatemia
Source: Front Pharmacol. 2026 Jul 29;17:1817481. doi: 10.3389/fphar.2026.1817481 (PMC13461917; doi:10.3389/fphar.2026.1817481)
Supplement: Supplementary file 1 [file Supplementaryfile1.docx]

**Table S1. Univariable logistic regression analysis of factors associated with AKI in sepsis patients.**

| **Variable** | **OR (95% CI)** | ***P* value** |
| --- | --- | --- |
| **Vancomycin parameters** |  |  |
| Initial VTC (mg/L) | 1.04 (1.02-1.07) | <0.001 |
| **Demographics** |  |  |
| Age (years) | 0.97 (0.96-0.99) | <0.001 |
| Gender (Female vs. Male) | 1.21 (0.78-1.88) | 0.385 |
| BMI (kg/m²) | 1.02 (0.99-1.04) | 0.195 |
| **Disease Severity** |  |  |
| SOFA score | 1.06 (0.99-1.14) | 0.120 |
| **Comorbidities** |  |  |
| Chronic kidney disease | 1.00 (0.57-1.77) | 0.988 |
| **Laboratory & Vital Parameters** |  |  |
| Albumin (g/dL) | 0.45 (0.28-0.72) | <0.001 |
| Lactate (mmol/L) | 1.13 (1.02-1.25) | 0.019 |
| Platelets (10⁹/L) | 1.00 (1.00-1.00) | 0.017 |
| **Treatments** |  |  |
| Vasopressor use | 1.54 (1.00-2.38) | 0.050 |
| Diuretic use | 1.64 (1.06-2.53) | 0.026 |

**Abbreviations:** AKI, acute kidney injury; OR, odds ratio; CI, confidence interval; VTC, vancomycin trough concentration; BMI, body mass index; SOFA, Sequential Organ Failure Assessment; CKD, chronic kidney disease. All variables were analyzed using univariate logistic regression. P values < 0.05 were considered statistically significant.

**Table S2. Multivariable logistic regression analysis of the association between initial vancomycin trough concentration (initial VTC) and acute kidney injury (AKI): multiple imputation analysis (N = 946).**

| **Exposure** | **Crude model OR (95% CI)** | ***P* value** | **Model 1 OR (95% CI)** | ***P* value** | **Model 2 OR (95% CI)** | ***P* value** |
| --- | --- | --- | --- | --- | --- | --- |
| Initial VTC (per 1 mg/L) | 1.07 (1.04-1.10) | <0.001 | 1.04 (1.02-1.07) | 0.002 | 1.03 (1.00-1.06) | 0.030 |
|  |  |  |  |  |  |  |
| **Initial VTC tertiles** |  |  |  |  |  |  |
| Low (≤10 mg/L) | Reference |  | Reference |  | Reference |  |
| Middle (>10 to ≤20 mg/L) | 2.54 (1.05-6.13) | 0.038 | 1.68 (0.91-3.12) | 0.099 | 1.51 (0.81-2.84) | 0.196 |
| High (>20 mg/L) | 4.41 (1.94-10.02) | <0.001 | 2.66 (1.49-4.74) | <0.001 | 2.19 (1.22-3.94) | 0.009 |

**Crude model:** unadjusted.

**Model 1:** adjusted for age, gender, and BMI.

**Model 2 (MI):** adjusted for age, gender, BMI, SOFA score, vasopressor use, CKD, albumin, lactate, platelet count, and diuretic use (10 covariates) using multiple imputation (5 imputations, n=946).

**Abbreviations:**  VTC, vancomycin trough concentration; AKI, acute kidney injury; OR, odds ratio; CI, confidence interval; BMI, body mass index; SOFA, Sequential Organ Failure Assessment; CKD, chronic kidney disease; MI, multiple imputation.

**Table S3. Threshold effect analysis of the relationship between the initial VTC and AKI using a two-piecewise regression model: Multiple Imputation Analysis. (N=946)**

| **Models** | **AKI OR (95%CI)** | ***P*-value** |
| --- | --- | --- |
| Model I |  |  |
| Linear effect | 1.03 (1.00, 1.06) | 0.030 |
| Model II |  |  |
| Knot (K) | 23.7 |  |
| Effect 1 (<K) | 1.05 (1.01, 1.10) | 0.019 |
| Effect 2 (>K) | 0.99 (0.92, 1.07) | 0.757 |
| Likelihood‑ratio test |  | 0.215 |

Data are presented as OR (95% CI). Model I: linear effect. Model II: two-piecewise regression with optimal knot (K = 23.7 mg/L) determined by minimizing AIC. Likelihood ratio test compares two-piecewise vs. linear model. Adjusted for age, gender, BMI, SOFA, vasopressor use, CKD, albumin, lactate, platelet count, and diuretic use (10 covariates) using multiple imputation (5 imputations, N = 946). Abbreviations: VTC, vancomycin trough concentration; AKI, acute kidney injury; OR, odds ratio; CI, confidence interval; AIC, Akaike information criterion.

**Table S4. Hospital clustering sensitivity analysis for the association between initial VTC and AKI.**

| **Variable** | **Standard Model OR (95% CI)** | **P value** | **Cluster-Robust OR (95% CI)** | **P value** |
| --- | --- | --- | --- | --- |
| initial_vtc_value | 1.06 (1.02-1.11) | 0.002 | 1.06 (1.02-1.11) | 0.002 |
| AGE | 0.96 (0.94-0.98) | <0.001 | 0.96 (0.94-0.98) | <0.001 |
| SOFA | 0.93 (0.83-1.05) | 0.233 | 0.93 (0.80-1.08) | 0.336 |
| albumin | 0.51 (0.29-0.90) | 0.020 | 0.51 (0.29-0.88) | 0.015 |
| Platelets | 1.00 (0.99-1.00) | 0.019 | 1.00 (0.99-1.00) | 0.009 |

All models adjusted for 10 covariates (age, gender, BMI, SOFA, vasopressor use, CKD, albumin, lactate, platelet count, diuretic use). Cluster-robust standard errors were calculated using the HC1 estimator, clustered by hospital ID (67 hospitals). Abbreviations: VTC, vancomycin trough concentration; AKI, acute kidney injury; OR, odds ratio; CI, confidence interval.

**Table S5. Sensitivity analysis with additional adjustment for GCS and APACHE IV scores**

| **Exposure** | **Crude model OR (95% CI)** | **P value** | **Model 1 OR (95% CI)** | **P value** | **Model 2 (Sensitivity) OR (95% CI)** | **P value** |
| --- | --- | --- | --- | --- | --- | --- |
| **Initial VTC (per 1 mg/L)** | **1.08 (1.04, 1.12)** | **<0.001** | **1.08 (1.04, 1.12)** | **<0.001** | **1.08 (1.04, 1.13)** | **<0.001** |
| **VTC tertiles** |  |  |  |  |  |  |
| Low (≤10 mg/L) |  |  |  |  |  |  |
| Middle (>10-20 mg/L) | 3.91 (1.44, 10.63) | 0.008 | 3.89 (1.41, 10.72) | 0.009 | 3.32 (1.14, 9.65) | 0.027 |
| High (>20 mg/L) | 8.40 (2.94, 24.01) | <0.001 | 8.22 (2.81, 24.03) | <0.001 | 7.82 (2.47, 24.76) | <0.001 |

Data are presented as OR (95% CI). Crude model: unadjusted. Model 1: adjusted for age, gender, and BMI. Model 2 (Sensitivity): adjusted for age, gender, BMI, SOFA score, vasopressor use, CKD, albumin, lactate, platelet count, diuretic use, GCS score, and APACHE IV score (12 covariates). N = 403, AKI events = 42. Abbreviations: VTC, vancomycin trough concentration; AKI, acute kidney injury; OR, odds ratio; CI, confidence interval; BMI, body mass index; SOFA, Sequential Organ Failure Assessment; CKD, chronic kidney disease; GCS, Glasgow Coma Scale; APACHE IV, Acute Physiology and Chronic Health Evaluation IV.

**Table S6. Threshold effect analysis (sensitivity analysis with GCS and APACHE IV)**

| **Models** | **Description** | **AKI OR (95% CI)** | **P-value** |
| --- | --- | --- | --- |
| **Model I** | **Linear effect** | **1.08 (1.04, 1.13)** | **<0.001** |
|  |  |  |  |
| **Model II** | **Knot (K) = 24** |  |  |
|  | Effect 1 (<24 mg/L) | 1.12 (1.04, 1.20) | 0.002 |
|  | Effect 2 (≥24 mg/L) | 1.02 (0.92, 1.14) | 0.664 |
|  | Likelihood ratio test |  | 0.227 |

**Table S7. Collinearity diagnostics for the 11 covariates**

|  | | |
| --- | --- | --- |
| **Variable** | **Variance Inflation Factor (VIF)** | **Interpretation** |
| Age (years) | 1.11 | No significant collinearity (<5) |
| Gender | 1.04 | No significant collinearity (<5) |
| Body mass index (kg/m²) | 1.04 | No significant collinearity (<5) |
| Vasopressor use | 1.15 | No significant collinearity (<5) |
| Chronic kidney disease | 1.02 | No significant collinearity (<5) |
| Albumin (g/dL) | 1.17 | No significant collinearity (<5) |
| Lactate (mmol/L) | 1.22 | No significant collinearity (<5) |
| Platelets (×10⁹/L) | 1.09 | No significant collinearity (<5) |
| Diuretic use | 1.11 | No significant collinearity (<5) |
| GCS score | 1.96 | No significant collinearity (<5) |
| APACHE IV score | 2.27 | No significant collinearity (<5) |

Note: Variance Inflation Factor (VIF) was calculated by regressing each covariate on all other covariates. Values below 5 indicate no significant multicollinearity. All VIF values were below 5 (range: 1.02–2.27), confirming the absence of significant collinearity among the covariates. Abbreviations: VIF, variance inflation factor; APACHE IV, Acute Physiology and Chronic Health Evaluation IV; GCS, Glasgow Coma Scale.

**Table S8. Baseline Severity Scores (GCS and APACHE IV) and Clinical Outcomes of Sepsis Patients Stratified by Initial Vancomycin Trough Concentration**

| **Characteristic** |  | **Initial VTC** | | | ***P* value** |
| --- | --- | --- | --- | --- | --- |
|  | **Entire population** | **Low Trough (≤10 mg/L)** | **Intermediate Trough (10-20 mg/L)** | **High Trough (>20 mg/L)** |  |
|  | **(N=946)** | **(N=337)** | **(N=424)** | **(N=185)** |  |
| **Exposure** |  |  |  |  |  |
| Initial VTC, mg/L | 12.85 (8.62-18.40) | 7.50 (6.00-8.90) | 14.20 (12.20-16.80) | 24.10 (21.90-28.40) | <0.001 |
| **Demographic data** |  |  |  |  |  |
| Age, years | 64.00 (53.00-74.00) | 63.00 (52.00-74.00) | 65.00 (54.00-75.00) | 62.00 (52.00-73.00) | 0.133 |
| Gender, n (%) |  |  |  |  | 0.590 |
| Female | 421 (44.5%) | 154 (45.7%) | 181 (42.7%) | 86 (46.5%) |  |
| Male | 525 (55.5%) | 183 (54.3%) | 243 (57.3%) | 99 (53.5%) |  |
| Ethnicity, n (%) |  |  |  |  | 0.736 |
| Caucasian | 716 (75.7%) | 244 (72.4%) | 329 (77.6%) | 143 (77.3%) |  |
| African American | 109 (11.5%) | 44 (13.1%) | 43 (10.1%) | 22 (11.9%) |  |
| Hispanic | 67 (7.1%) | 31 (9.2%) | 26 (6.1%) | 10 (5.4%) |  |
| Asian | 18 (1.9%) | 6 (1.8%) | 9 (2.1%) | 3 (1.6%) |  |
| Native American | 15 (1.6%) | 4 (1.2%) | 7 (1.7%) | 4 (2.2%) |  |
| Other/Unknown | 21 (2.2%) | 8 (2.4%) | 10 (2.4%) | 3 (1.6%) |  |
| BMI, kg/m² | 26.58 (22.38-31.87) | 24.51 (21.22-29.82) | 26.62 (22.77-31.48) | 29.24 (24.62-36.53) | <0.001 |
| Severity score |  |  |  |  |  |
| SOFA score | 4.00 (2.00-6.00) | 4.00 (2.00-6.00) | 4.00 (2.00-6.00) | 5.00 (3.00-7.00) | 0.004 |
| GCS score | 13.00 (9.00-15.00) | 13.00 (9.00-15.00) | 13.00 (9.00-15.00) | 11.00 (8.00-15.00) | 0.010 |
| APACHE IV score | 68.50 (53.00-87.00) | 68.00 (51.00-85.25) | 69.00 (55.00-86.00) | 70.00 (54.00-87.00) | 0.371 |
| Comorbidities |  |  |  |  |  |
| Chronic kidney disease, n (%) | 164 (17.3%) | 63 (18.7%) | 69 (16.3%) | 32 (17.3%) | 0.681 |
| **Sepsis source of infection** |  |  |  |  | *0.210* |
| *Pulmonary* | *466 (49.3%)* | *180 (53.4%)* | *206 (48.6%)* | *80 (43.2%)* |  |
| *Renal/UTI* | *156 (16.5%)* | *52 (15.4%)* | *67 (15.8%)* | *37 (20.0%)* |  |
| *GI* | *89 (9.4%)* | *28 (8.3%)* | *40 (9.4%)* | *21 (11.4%)* |  |
| *Cutaneous/soft tissue* | *104 (11.0%)* | *35 (10.4%)* | *47 (11.1%)* | *22 (11.9%)* |  |
| *Gynecologic* | *1 (0.1%)* | *0 (0.0%)* | *0 (0.0%)* | *1 (0.5%)* |  |
| Laboratory values |  |  |  |  |  |
| Albumin, g/dL | 2.40 (2.00-2.80) | 2.50 (2.10-2.90) | 2.40 (2.00-2.70) | 2.30 (1.80-2.70) | <0.001 |
| Lactate, mmol/L | 1.60 (1.10-2.60) | 1.65 (1.08-2.60) | 1.60 (1.10-2.50) | 1.70 (1.20-2.80) | 0.578 |
| Platelets, ×10⁹/L | 188.00 (121.00-269.25) | 171.00 (119.00-264.00) | 196.00 (125.00-280.00) | 188.00 (116.25-259.75) | 0.116 |
| Nephrotoxic medications |  |  |  |  |  |
| Vasopressor use, n (%) | 343 (36.3%) | 120 (35.6%) | 140 (33.0%) | 83 (44.9%) | 0.019 |
| Diuretic use, n (%) | 440 (46.5%) | 145 (43.0%) | 202 (47.6%) | 93 (50.3%) | 0.233 |
| Aminoglycoside use, n (%) | 3 (0.3%) | 2 (0.6%) | 1 (0.2%) | 0 (0.0%) | 0.604 |
| Piperacillin-tazobactam use, n (%) | 343 (36.3%) | 120 (35.6%) | 158 (37.3%) | 65 (35.1%) | 0.840 |
| Nitroimidazole use, n (%) | 81 (8.6%) | 35 (10.4%) | 28 (6.6%) | 18 (9.7%) | 0.147 |
| ACEI/ARB use, n (%) | 77 (8.1%) | 36 (10.7%) | 35 (8.3%) | 6 (3.2%) | 0.012 |
| NSAID use, n (%) | 49 (5.2%) | 24 (7.1%) | 16 (3.8%) | 9 (4.9%) | 0.115 |
| Outcomes |  |  |  |  |  |
| AKI occurrence, n (%) | 92 (9.7%) | 22 (6.5%) | 42 (9.9%) | 28 (15.1%) | 0.006 |
| 28-day mortality, n (%) | 74 (7.8%) | 18 (5.3%) | 29 (6.8%) | 27 (14.6%) | <0.001 |

Data are presented as mean ± SD or median (IQR) based on normality test. Group comparisons: Kruskal-Wallis test for continuous variables, Chi-square/Fisher's exact test for categorical variables.

**Abbreviations**: AKI, acute kidney injury; BMI, body mass index; CKD, chronic kidney disease; IQR, interquartile range; SOFA, Sequential Organ Failure Assessment; VTC, vancomycin trough concentration.

**Missing data**: BMI (1.5%), albumin (27.6%), lactate (36.0%), platelets (2.7%), APACHE IV (9.5%), GCS (3.2%).

**Table S9. Sensitivity analysis: Multivariable logistic regression analysis of the association between initial vancomycin trough concentration and severe AKI**

| **Exposure** | **Crude model OR (95% CI)** | **Crude P** | **Model 1 OR (95% CI)** | **Model 1 P** | **Model 2 OR (95% CI)** | **Model 2 P** |
| --- | --- | --- | --- | --- | --- | --- |
| **Initial VTC (per 1 mg/L)** | **1.07 (1.02, 1.12)** | **0.003** | **1.04 (1.00, 1.09)** | **0.068** | **1.06 (1.00, 1.12)** | **0.036** |
|  |  |  |  |  |  |  |
| **VTC tertiles** |  |  |  |  |  |  |
| Low (≤10 mg/L) |  |  |  |  |  |  |
| Middle (>10 to ≤20 mg/L) | 2.01 (0.47, 8.54) | 0.347 | 1.03 (0.34, 3.15) | 0.954 | 1.76 (0.40, 7.83) | 0.456 |
| High (>20 mg/L) | 4.19 (1.15, 15.32) | 0.030 | 1.92 (0.74, 5.00) | 0.182 | 3.00 (0.77, 11.64) | 0.113 |

**Abbreviations:** VTC, vancomycin trough concentration; AKI, acute kidney injury; OR, odds ratio; CI, confidence interval; BMI, body mass index; SOFA, Sequential Organ Failure Assessment; CKD, chronic kidney disease.

**Model 1:** Adjusted for age, gender, and BMI.

**Model 2:** Adjusted for age, gender, BMI, SOFA score, vasopressor use, CKD, albumin, lactate, platelet count, and diuretic use (10 covariates).

**Table S10. Sensitivity analysis: Threshold effect analysis of the relationship between initial vancomycin trough concentration and severe AKI using a two-piecewise regression model**

| **Models** | **Description** | **AKI OR (95% CI)** | **P-value** |
| --- | --- | --- | --- |
| **Model I** | **Linear effect** | **1.06 (1.00, 1.12)** | **0.036** |
|  |  |  |  |
| **Model II** | **Knot (K) = 6.5** |  |  |
|  | Effect 1 (<6.5 mg/L) | 0.63 (0.34, 1.17) | 0.145 |
|  | Effect 2 (≥6.5 mg/L) | 1.08 (1.01, 1.14) | 0.014 |
|  | Likelihood ratio test |  | 0.152 |

**Abbreviations:** VTC, vancomycin trough concentration; AKI, acute kidney injury; OR, odds ratio; CI, confidence interval.

**Model I:** Linear model assuming a constant effect of VTC on severe AKI risk across the entire range of VTC values.

**Model II:** Two-piecewise regression model with an optimal threshold (knot) determined by minimizing the Akaike information criterion (AIC). The search range for the optimal knot was 5.0-24.5 mg/L (10th to 90th percentiles of VTC distribution).

**Adjustment:** Both models were adjusted for age, gender, BMI, SOFA score, vasopressor use, CKD, albumin, lactate, platelet count, and diuretic use (10 covariates).
